# A novel technique of harmonic tissue dissection reduces seroma formation after modified radical mastectomy compared to conventional electrocautery: a single-blind randomized controlled trial

**DOI:** 10.1186/s13037-018-0155-3

**Published:** 2018-05-17

**Authors:** Mohammed Faisal, Hamada Fathy, Hamdy Shaban, Sameh T. Abuelela, Ahmed Marie, Islam Khaled

**Affiliations:** Surgical Oncology Unit, Department Of Surgery, Faculty of Medicine, Suez Canal University, Circular Road, Ismailia, 411522 Egypt

## Abstract

**Background:**

Seroma is the most frequent postoperative complication following breast cancer surgery. Our aim was to evaluate the effect of the harmonic focus scalpel versus electrocautery in reducing seroma formation post-mastectomy and axillary clearance.

**Methods:**

A prospective randomized controlled trial study was conducted at the Department of Surgery of Suez Canal University Hospital from April 26th 2014 to 30th June 2016. Seventy-two women, in whom a mastectomy and axillary clearance for breast cancer were performed, were randomly allocated to either harmonic dissection (*n* = 36) or electrocautery (*n* = 36).

**Results:**

The mean operative time was significantly longer for harmonic dissection compared with electrocautery (2.63 ± 0.41 vs. 1.75 ± 0.26 h; *p* < 0.0001). In addition, a significantly smaller amount of intraoperative blood loss (69.4 ± 25.1 vs. 255.5 ± 41.6 ml; *p* = 0.002) and total drainage volume (1277.8 ± 172.5 ml vs. 3300 ± 167.5 ml; *p* = 0.002) were found in the harmonic group. Moreover, there was a significant reduction in the time of drain removal (10.9 ± 1.12 vs. 15.9 ± 1.44; *p* = 0.001) and the incidence of seroma formation after drain removal [8.3% vs 33.3%; *p* = 0.003] in the harmonic group compared with those in the electrocautery group.

**Conclusion:**

Harmonic dissection technique leads to significant decreases in intraoperative blood loss, total drainage volume and postoperative seroma in terms of shorter drain duration with a minimal increase in the operative time and better quality of life. Here, we recommend the use of the harmonic dissection technique in mastectomy and axillary clearance.

## Background

Seroma is the most frequent postoperative complication following breast cancer surgery, with an incidence rate ranging between 15% and 85% [1]. Seroma occurs in the axilla, triggering pain and limiting arm movements. Wound seroma may lead to late drain removal and increased long-term morbidity and can also lead to flap necrosis, wound dehiscence, postponed recovery and delayed adjuvant therapy [2].

Electrocautery has been widely used for resection purposes in breast surgery, as it significantly reduces blood loss compared with conventional scalpel use; nevertheless, the use of electrocautery may increase the occurrence of seroma following modified radical mastectomy (MRM), as it causes thermal tissue damage in the skin flaps, leading to local inflammatory reaction, subdermal vascular plexus disruption, and incomplete lymphatic and vascular occlusion; these complications often contribute to a higher seroma morbidity rate [3].

Ultrasonic wave technology enables blood vessels less than 5 mm in diameter to be sealed in the coagulation mode and has the ability to dissect and create flaps with minimal extensive thermal damage that will not exceed 1.5 mm, making ultrasound dissection devices preferred by many surgeons as harmonic scalpels [4]. The harmonic scalpel has been extensively used in laparoscopic surgery for surgical dissection based on findings from its use in open surgery, namely, that it can significantly reduce blood loss and operative time [5].

Compared with electrocautery, harmonic dissection has several advantages, including less scar formation compared with blades, the narrow circumference of collateral thermal damage to encircling tissue, the lack of smoke (although there is a transient mist), the lack of injury or excitement to motor nerves in the axilla, and the ability to utilize the technique in patients with pacemakers [6].

The aim of this study was to evaluate the effect of the harmonic focus scalpel versus electrocautery in reducing seroma formation post-MRM.

## Methods

### Study design

**Fig. 1:**
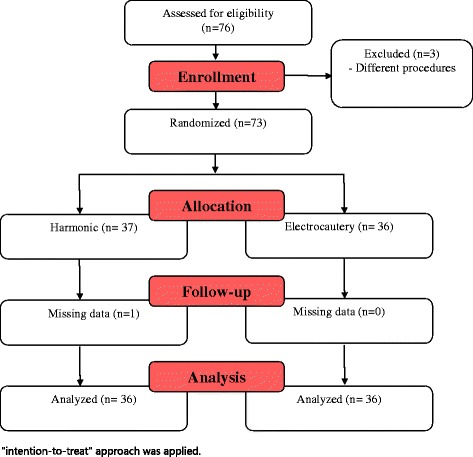
Flow diagram of participants

This study was a single-blind randomized controlled trial that took place at the Surgical Oncology Unit of the Department of Surgery, Suez Canal University Hospital, from 26th April 2014 to 30th June 2016 comparing the clinical outcomes of using harmonic dissection technique using Ethicon Generator G11 and harmonic focus handle (Ethicon Endo-Surgery, Inc., NJ, USA) versus electrocautery technique. This research was reviewed by the Research Ethics Committee of the Faculty of Medicine at Suez Canal University at its meeting on 23/4/2014 (reference number: #2132). Written and verbal informed consent was obtained from the selected patients (Fig. 1).

### Study population

We assessed the eligibility of all women with a diagnosis of operable breast cancer who underwent modified radical mastectomy were included in the study. We excluded patients with immediate reconstruction, were scheduled for other simultaneous procedure, had recurrent breast cancer patient, with previous radiation over chest wall, who cannot understand or cannot accept the study (refused to sign written informed consent) or patient unfit for surgery.

### Study hypothesis

We hypothesised that the usage of harmonic dissection technique in modified radical mastectomy will decrease the incidence of seroma formation and other post-operative complications.

### Sampling and randomization

The sample size calculation was based on historical data from our centre regarding the seroma rate in elective mastectomy and axillary clearance (24%) and an expected seroma rate of 9% in the harmonic scalpel group [7] based on the best data reported in the literature on the seroma rate after elective MRM. Based on an 80% power and a significance level of 0.05, 36 patients were required in each arm of the study. The number of patients was increased by 20% in anticipation of loss at follow-up.

A random sequence was generated using the random function of the Microsoft Excel programme. The surgeon was given the randomly generated treatment allocations within sealed opaque envelopes. Once a patient consented to enter the trial, the envelope was opened, and the patient underwent the allocated surgery. Thirty-sex patients were equally allocated with ratio 1:1 and randomly assigned to each of the study and control groups.

Our primary analysis was conducted using an intent-to-treat approach, and patients were analyzed in their groups as they were after randomization.

### Study outcomes

- The primary outcome was to identify seroma formation in both study groups. Seroma was defined as presence of fluid collection beneath the skin flaps after the removal of the drains of sufficient quantity to cause the patient discomfort and was measured by subcutaneous aspiration and US during the postoperative follow up (30 days).
- Secondary outcomes were recorded as
  - intraoperative measures;
    - Operative time: from skin incision to skin closure.
    - Blood loss estimated by suction gar and sponge through weighing the dry sponges preoperatively and subtracting the weight from the weight of the used sponges.
  - Postoperative measures:
    - Total drainage volume in ml.
    - Time of drain removal.
    - Wound complications [haematoma, wound infection, delayed wound healing and flap necrosis].

### Statistical analysis

Statistical analysis was performed using the statistical software SPSS 23.0 (SPSS Inc., Chicago, IL, USA). Quantitative variables that followed a normal distribution are reported as the means and standard deviations. For non-Gaussian variables, the median and range were used. Qualitative variables are reported as numbers and percentages of cases.

Quantitative variables were compared using Student’s t test, and the chi-square test was also used for qualitative variables. In cases with fewer than 5 observations in the cell, Fisher’s exact probability method was used. *P* < 0.05 was regarded as significant.

### Preoperative workup

All the studied patients were subjected to full history taking, general and local clinical examination, and preoperative evaluation of the tumor regarding its extent and the presence of distant metastasis. The investigations included ultrasonography and soft tissue mammography of both breasts and axillae, preoperative needle biopsy (either fine needle aspiration cytology or Tru-Cut needle biopsy) and a metastatic workup (chest X-ray, abdominal and pelvic ultrasonography and bone gammagraphy if necessary).

### Study intervention and surgical procedure

**Fig. 2:**
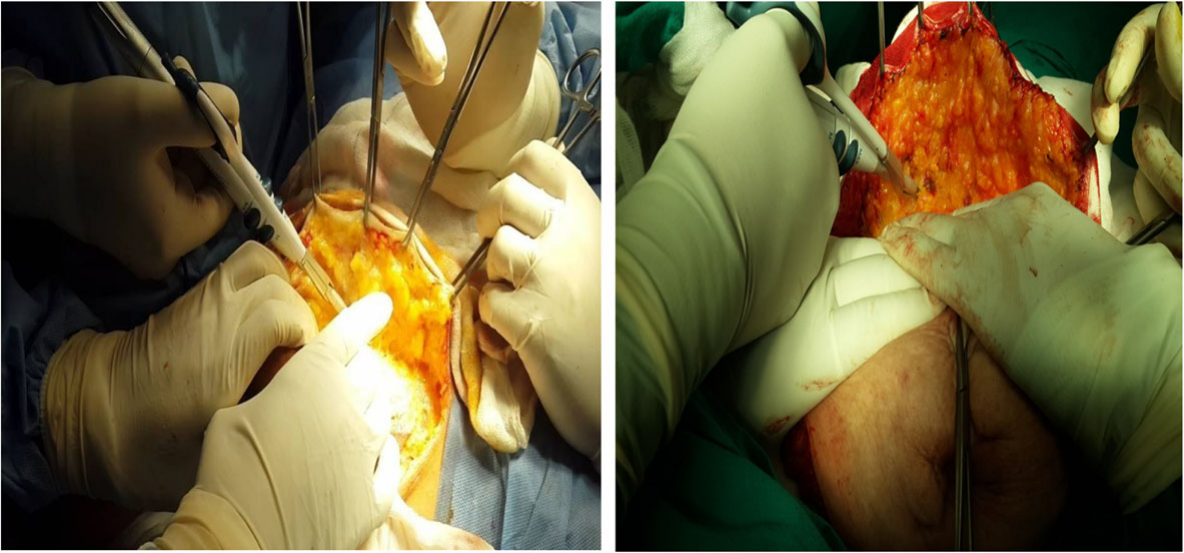
Showing bloodless field in raising skin flaps in MRM using harmonic focus scalpel

**Fig. 3:**
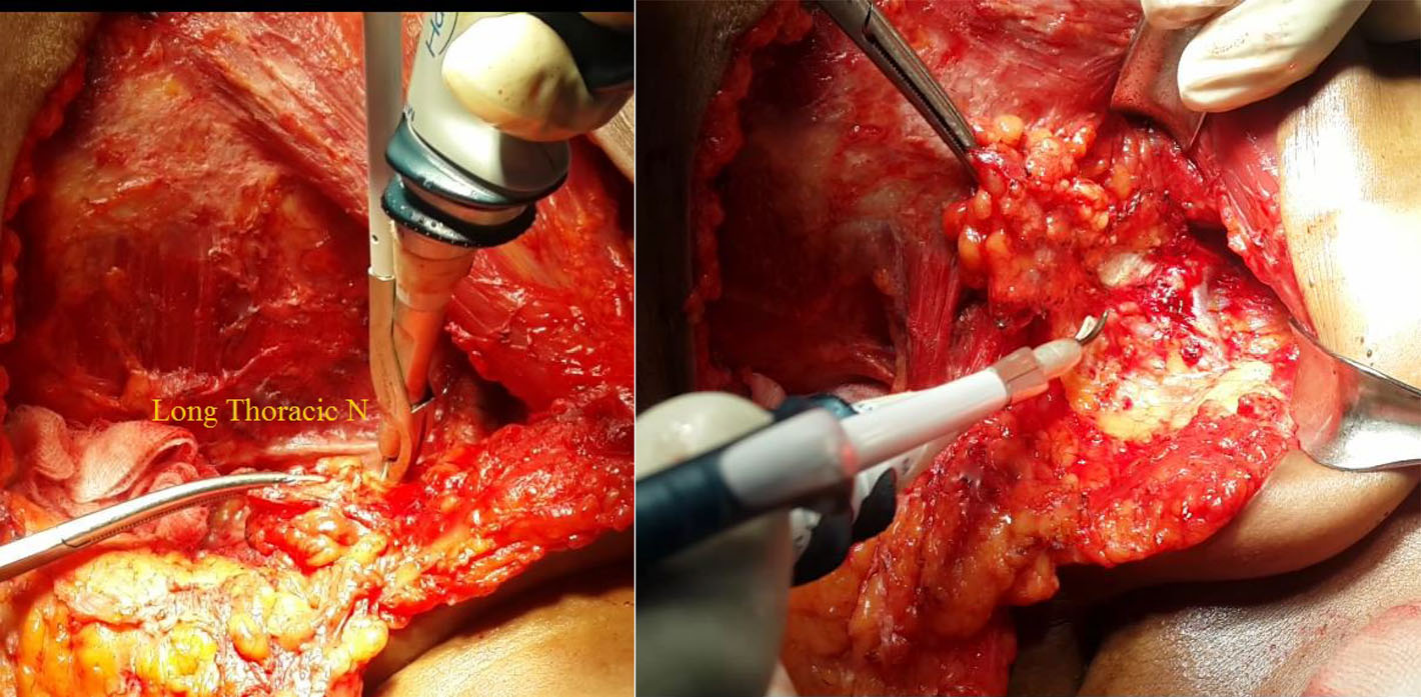
Showing axillary lymph node dissection using harmonic focus

All patients were operated on by a single surgeon with experience using the harmonic scalpel technique and the same surgical team, and the skin incision was made using a conventional scalpel. Upper and lower skin flaps were raised, and the breast tissue with the pectoral fascia was reflected off the pectoralis major muscle using electrocautery or the harmonic dissection technique according to the randomization. The clavipectoral fascia was opened, and the medial and lateral borders of the pectoralis minor were defined. The pectoralis minor muscle was retracted, and the axilla was exposed. The axillary vein was identified, and all its small tributaries were dissected. Axillary lymph node dissection was initiated from the lateral end of the vein. A plane of dissection was created along the inferior border of the axillary vein. The lymph nodes and blood vessels were dissected off the axillary vein towards the breast. The thoracodorsal bundle and the long thoracic, subscapular, medial and lateral anterior nerves were defined and protected. Level I and II axillary lymph node dissections were performed in all patients, and level III dissection was possibly included if the axillary lymph nodes were grossly involved. The previous dissection, lymph vessel sealing and haemostasis were performed using the harmonic device or electrocautery according to the randomization. The surgical field was douched with normal saline, and two 18-F suction drains were inserted: one in the axilla and the other under the skin flaps. The wound was closed using sutures (Figs. 2 and 3).

### Follow-up

All patients were discharged 24 h after surgery with drains. A card was given to each patient at the time of discharge to record the drain volume at home daily at a specified time of the day, and the volume was measured after placing the bottle on a flat surface. Patients were followed up in the outpatient clinic until drain removal with instructions to return to the clinic early if the drain bottle was filled up, if leakage from around the drain was encountered, or if the drain bottle’s vacuum was lost. The drains were removed when the drainage volume was less than 30 ml over 24 h for 2 successive days.

## Results

A total of 76 patients were included in the study. Three patients were excluded from the analysis because they underwent a different procedure. One patient in Group 1 was lost to follow-up during the postoperative course. In total, 36 patients were analyzed in Group 1 and 36 were analyzed in Group 2 using an intent-to-treat approach, and patients were analyzed in their groups as they were after randomization (Flow chart 1).

The patient sample consisted of 72 female patients. Patient age ranged between 42 and 65 years (mean age 51.8 years) in the harmonic group and between 42 and 68 years (mean 52.5 years) in the electrocautery group. Family history was positive in 11.1% of the patients in the harmonic group and in 22.2% of patients in the electrocautery group. Of the patients in the harmonic group, 11.1% were nullipara, while only 5.6% of the patients in the electrocautery group were nullipara. A total of 75% of the patients had stage II breast cancer in study groups with 26 patients (72.2%) in harmonic Group and 30 patients (83.3%) in electrocautery group. Regarding modern AJJC breast cancer staging in both groups, 58.3% of patients in harmonic group were stage IIB, this percent was increased to 63.9% in electrocautery group. Both groups were comparable in terms of their sociodemographic data, as shown in Table 1.

**Table 1:** Baseline variables between harmonic and electrocautery groups

Variable	Harmonic (n = 36)	Electrocautery (n = 36)	p
- Age (years)	51.8 ± 6.5	52.5 ± 7.8	0.707^a^
- BMI	29.5 ± 3.1	29.6 ± 5.4	0.206 ^a^
- Comorbidities			
HTN	16 (44.4%)	18 (50%)	0.317 ^b^
DM	12 (33.3%)	14 (38.9%)	0.523 ^b^
CLD	11 (30.6%)	13 (36.1%)	0.628 ^b^
- Smoking	5 (13.9%)	3 (8.3%)	0.427 ^b^
- Family history			
Positive	4 (11.1%)	8 (22.2%)	0.316 ^b^
- Parity			
Nullpara	4 (11.1%)	2 (5.6%)	0.716 ^b^
Multipara	32 (88.9%)	34 (94.4%)	0.521 ^b^
- Breast Weight (G)	1511 ± 584.5	1641 ± 828.6	0.139 ^a^
- Clinical TNM stage			
Stage I	4 (11.1%)	2 (5.6%)	0.637 ^b^
Stage II	26 (72.2%)	30 (83.3%)	
Stage III	6 (16.7%)	4 (11.1%)	
-AJCC tumor staging			
Stage I A	1 (2.8%)	1 (2.8%)	0.327 ^b^
Stage I B	3 (8.3%)	1 (2.8%)	
Stage II A	5 (13.9%)	7 (19.4%)	
Stage II B	21 (58.3%)	23 (63.9%)	
Stage III A	5 (13.9%)	2 (5.5%)	
Stage III B	1 (2.8%)	2 (5.5%)	
- No. of lymph nodes retrieved ^d^	21 (11 – 32)	17 (10 – 27)	0.262^c^
- Neoadjuvant therapy	5 (13.9%)	6 (16.7%)	0.745 ^b^
- Histology			
Ductal carcinoma	34 (94.4%)	32 (88.9%)	0.243 ^b^
Lobular	2 (5.6%)	4 (11.1%)	

^a^t test

^b^χ^2^ test

^c^Mann–Whitney U test

^d^Median (interquartile range)

Harmonic dissection yielded significantly better outcomes (Table 2) than electrocautery. Specifically, significant *p* values were obtained from the t test for intraoperative blood loss (69.4 ± 25.1 vs 255.5 ± 41.6 ml, *p* < 0.002), total drainage volume in ml (1277.8 ± 172.5 vs 3300 ± 167.5, *p* < 0.002), time of drain removal in days (13.3 ± 1.13 vs 17.9 ± 1.64, *p* < 0.001), and overall seroma (8.3% vs 33.3%, *p* < 0.003), and operative time in hours (2.63 ± 0.41 vs 1.75 ± 0.26, *p* < 0.0001. Ultrasonic wave dissection using the harmonic device is better than electrocautery using monopolar diathermy in terms of overall complications [5 (13.88%) vs 14 (3.88%)]. Our study demonstrated numerical reductions in event rates of postoperative wound complication such as postoperative hematoma which was less in harmonic group than electrocautery group [2 (5.6%) vs 7 (19.4%)], also wound infection and wound healing were noticed in 5.6% of patients in harmonic group and this percent was increased to 22.2% in electrocautery group while there was no cases in harmonic group suffered from flaps ischemia and 27.8% of cases in electrocautery group suffered from flaps necrosis although not statistically significant except in postoperative seroma formation after drain removal *p* < 0.003.

**Table 2:** Comparison of outcome variables between harmonic and electrocautery groups

Variable	Harmonic (n = 36)	Electrocautery (n = 36)	p
- Duration of surgery (hours)	2.63± 0.41	1.75 ± 0.26	0.0001 ^a^
- Blood loss (ml)	69.4 ± 25.1	255.5 ±41.6	0.002 ^a^
- Total drain volume (ml)	1277.8 ± 172.5	3300 ±167.5	0.002 ^a^
- Duration of drains (days)	13.3 ± 1.13	17.9 ± 1.64	0.001 ^a^
- Complications			
Wound infection	2 (5.6%)	8 (22.2%)	0.569 ^b^
Delayed Healing	2 (5.6%)	8 (22.2%)	0.569 ^b^
Flap necrosis	0	5 (27.8%)	0.435 ^b^
Hematoma	2 (5.6%)	7 (19.4%)	0.164 ^b^
Re-accumulation of seroma after drain removal	3 (8.3%)	12 (33.3%)	0.003 ^b^

^a^t test

^b^χ^2^ test

The time of drain removal in the harmonic group was 4.6 days less than that in the electrocautery group after adjusting for the variables of breast weight, body mass index (BMI) and number of lymph nodes excised from the specimen (r2 = 0.32, β = 12.3, *p* < 0.001).

Overall seroma in the harmonic group was less than that in the electrocautery group [ARR 0.036 (95% CI 0.002–0.21)] after adjusting for age, breast weight, neoadjuvant chemotherapy and BMI (Table 3). Similarly, the risk of postoperative complications and comorbidities in the harmonic group was significantly lower than that in the electrocautery group [ARR 0.43 (95% CI 0.23–0.84)] after adjusting for neoadjuvant therapy and BMI (Table 4).

**Table 3:** Univariate and multivariable regression analysis for significant postoperative re-accumulation of seroma after drain removal

Covariate	Univariate CRR (95 % CI)	Multivariate ARR (95 % CI)
- Intervention		
Electrocautery	1	1
Harmonic	0.032 (0.001 – 0.17)	0.036 (0.002 – 0.21)
- Age		
Up to 55	1	1
≥ 56	1.2 (0.65 – 2.14)	1.41 (0.74 – 2.56)
- Weight of specimen		
Up to 1000 g	1	1
≥ 1000 g	0.72 (0.41 – 1.56)	0.94 (0.57 – 1.98)
- Neoadjuvant therapy		
No	1	1
Yes	1.1 (0.78 – 2.02)	1.3 (0.84 – 2.34)
- BMI		
Normal	1	1
Overweight	0.71 (0.38 – 1.68)	0.76 (0.42 – 1.71)
Obese	0.87 (0.49 – 1.96)	0.94 (0.65 – 2.07)

*CRR* crude relative risk, *CI* confidence interval, *ARR* adjusted relative risk

**Table 4:** Univariate and multivariable regression analysis for overall complications

Covariate	Univariate CRR (95 % CI)	Multivariate ARR (95 % CI)
- Intervention		
Electrocautery	1	1
Harmonic	0.48 (0.25 – 0.88)	0.43 (0.23 – 0.84)
- Neoadjuvant therapy		
No	1	1
Yes	0.77 (0.46 – 1.51)	0.71 (0.41 – 1.46)
- BMI		
Normal	1	1
Overweight	0.89 (0.47 – 1.95)	0.87 (0.45 – 1.92)
Obese	1.15 (0.52 – 2.56)	1.07 (0.49 – 2.41)

*CRR* crude relative risk, *CI* confidence interval, *ARR* adjusted relative risk

## Discussion

Seroma is the most frequent postoperative complication following breast cancer surgery [1]. Electrocautery has been widely used for dissection purposes in breast surgery because it significantly reduces blood loss compared with conventional scalpel use; nevertheless, the use of electrocautery may increase the occurrence of seroma following MRM.

The harmonic dissection technique is currently emerging as an alternative surgical tool for dissection and haemostasis and is thought to reduce morbidities such as seroma and blood loss. Compared with electrocautery, harmonic dissection has several advantages, including lack of scar formation, narrow circumference of collateral thermal damage to encircling tissue, lack of smoke (although there is a transient mist), lack of injury or excitement to motor nerves in the axilla, and ability to utilize the technique in patients with pacemakers [8]. Generally, the use of harmonic dissection has proven beneficial in a variety of surgeries; however, its role in breast surgery remains controversial.

Operative time appears longer with the harmonic dissection technique than that with electrocautery [9]. In our study, the operative time required for MRM and axillary dissection was significantly longer in patients operated on by the harmonic scalpel compared with electrocautery (2.63 h vs. 1.75 h, respectively). These results are consistent with those of a study conducted by Abul Nagah et al. in Egypt in forty patients with operable breast cancer who reported that the operative time was longer in their first group to undergo surgery using the harmonic scalpel because it was a new dissection device and required an adaptation period [9]. Deo et al. in Singapore, also found that the mean operative time was longer in the harmonic scalpel patients [10]. However, our results contrast with those of a study conducted by Khater in Egypt in sixty females undergoing MRM using the harmonic scalpel or electrocautery, which showed no significant difference between the two groups regarding operative time [11]. Sanguinetti et al. compared the use of the harmonic scalpel with electrocautery for axillary dissection and noted that there was no significant difference in the operative time [12].

Our study revealed that the use of the harmonic dissection technique significantly reduces intraoperative blood loss compared with electrocautery (69.4 ml vs. 255.5 ml, respectively). These results are concordant with many studies, including that conducted by Abul Nagah et al. who reported that the mean operative blood loss was significantly less in the group that underwent surgery with the harmonic scalpel compared with that in the group that underwent surgery using electrocautery [9]. In addition, Deo et al. found that the haemostatic power of the harmonic scalpel was better than that of electrocautery [10].

Our study demonstrated numerical reductions in event rates of postoperative wound complications including postoperative hematoma, wound infection, delayed wound healing and flab necrosis although not statistically significant. The incidence of wound infection was higher among patients in the electrocautery group (22.2%) compared with that in the patients in the harmonic group (5.6%), and the same percentages for delayed wound healing with no statistical significance. However, no patients in the harmonic group suffered from flap ischemia, while 27.8% of the patients in the electrocautery group suffered from flap ischemia and necrosis. Postoperative hematoma which was less in harmonic group than electrocautery group (5.6% vs 19.4%).

Our results were similar to those reported by Ribeiro et al. who found a lower incidence of overall complications associated with harmonic dissection compared with that associated with electrocautery in MRM (29% vs. 52%, respectively) [13].

Yilmaz et al. [14], found the same results regarding the post-operative wound complication as they showed that there was no statistical difference between both group with respect to hematoma, wound infection and flap necrosis with reduced events rat in harmonic group than electrocautery group.

Khan et al. [15] also reported that the incidence of postoperative wound complications as hematoma, surgical site infection and flap necrosis was higher in the diathermy group. Khater [11] reported in his study that there was no statistical difference between the two groups regarding flap necrosis (*P* = 1). Kozomara et al. [7] also reported that there was no statistical difference between the two groups regarding types of postoperative complications as wound infection or wound dehiscence. Damani et al. [6] also stated in a study conducted on fifty female patients underwent MRM in Pakistan that there was no statistical difference in the use of both techniques in terms of hematoma (*p* = 0.235), flap necrosis (*p* = 1.000) and lymphedema (*p* = 1.000).

These results can be explained that electrocautery has high thermal energy that can result in the devitalization of tissues and the ability of the harmonic technique dissection to provide proper blood vessels sealing with less risk of thermal damage, as it operates at lower temperatures, whereby less energy is dispersed to nearby and deeper tissues [16].

Other advantages found in our study regarding the use of the harmonic technique dissection included significant reductions in total drainage volume (1277.8 ml vs. 3300 ml) and time of drain removal (10.9 vs. 15.9 days) and a lower incidence of seroma formation after drain removal (8.3% vs. 33.3%). These results can be explained by the ability of the harmonic technique dissection to provide better haemostasis with less lateral thermal injury; thus, undesirable extra injury is avoided when the harmonic technique dissection is used. Moreover, the inflammatory reaction in the operative field is reduced [14]; fewer lymphatic vessels are injured, with proper sealing for injured lymphatic vessels; and fewer oozing surfaces are produced in the operative field. Together, these factors reduce the postoperative drainage volume and, consequently, may reduce the postoperative hospital stay. Our results are similar to those reported by Abul Nagah et al. who stated that the mean total drainage volume in their harmonic group was significantly lower than that in their electrocautery group (*p* = 0.002) [9]. Similar results were reported by Deo et al. and Galatius et al. [10, 17]. Khater reported that the use of the harmonic scalpel significantly reduced the total amount of drainage fluid and number of drainage days (*p* < 0.001) between the two groups 10.

In our study, the harmonic technique dissection significantly decreased the rate of re-occurrence of seroma after drain removal compared with electrocautery (8.3% vs 33.3%, respectively; *p* < 0.003). Similar results were reported by Anlar et al. (2012) and another trial, which focused mainly on conservative breast surgery and revealed a significant reduction in seroma formation [16, 18].

### Study limitations

Our study was a single-blinded study, as we could not blind the surgical team to the intervention, and we used two different tools in the surgical procedure. Our study reported a lower incidence of axillary seroma in harmonic dissection compared with electrocautery in one breast surgery procedure, MRM, and we did not study its benefit in other breast procedures, such as conservative breast surgery and simple mastectomy, because mastectomy is preferred over breast conservation in our country due to the presence of advanced disease at the time of presentation and a lack of resources for long-term follow-up after conservative breast surgery. In addition, the sample size was not sufficiently large to validate these findings; therefore, we recommend a further multicentre trial that includes a larger sample and multiple procedures to compare subtle differences between individual complications, which would broaden the generalizability of the results. Estimating intraoperative blood loss can be a difficult task, especially when blood is mostly absorbed by gauze. Bleeding from both groups was not clinically significant and the method used in our study to estimate bleeding amount was the best way to estimate bleeding as it is much criticized because the towels absorb fluids as a false estimate of amount of bleeding.

## Conclusions

In summary, harmonic dissection leads to significant decreases in intraoperative blood loss, total drainage volume and postoperative discomfort. Additionally, harmonic dissection results in a shorter duration before drain removal and a decreased incidence of seroma formation, with a minimal increase in the operative time. Because these are important postoperative care factors, we recommend the harmonic dissection technique for use in MRM.

## Abbreviations

AJJC: American joint committee on cancer
ARR: Adjusted relative risk
BMI: Body Mass Index
CI: Confidence interval
CRR: Crude relative risk
MRM: Modified radical Mastectomy
US: Ultrasound

## References

[1] van Bemmel AJM, van de Velde CJH, Schmitz RF, Liefers GJ (2011). Prevention of seroma formation after axillary dissection in breast cancer: a systematic review. Eur J Surg Oncol J Eur Soc Surg Oncol Br Assoc Surg Oncol.

[2] Kuroi K (2006). Evidence-based risk factors for seroma formation in breast surgery. Jpn J Clin Oncol.

[3] Shamim M (2009). Diathermy vs. scalpel skin incisions in general surgery: double-blind, randomized, clinical trial. World J Surg.

[4] Siperstein AE, Berber E, Morkoyun E (2002). The use of the harmonic scalpel vs conventional knot tying for vessel ligation in thyroid surgery. Arch Surg Chic Ill 1960.

[5] Fried G (2001). Hemostatic tools for the gastrointestinal surgeon: ultrasonic coagulator vs. bipolar ligation. J Gastrointest Surg.

[6] Damani SR, Haider S, Shah SSH (2013). Comparison of modified radical mastectomy using harmonic scalpel and electrocautery. J Surg Pak Int.

[7] Kozomara D, Galić G, Brekalo Z, Šutalo N, Kvesić A, Šoljić M (2010). A randomised two-way comparison of mastectomy performed using harmonic scalpel or monopolar diathermy. Coll Antropol.

[8] Clymer J (2016). A systematic review and meta-analysis of harmonic technology compared with conventional techniques in mastectomy and breast-conserving surgery with lymphadenectomy for breast cancer. Breast Cancer Targets Ther.

[9] Tarek A ANG, Tarek EF LH, Shehab W (2007). Comparative study between using harmonic scalpel and electrocautery in modified radical mastectomy. Egypt J Surg.

[10] Deo SVS, Shukla NK, Asthana S, Niranjan B, Srinivas G (2002). A comparative study of modified radical mastectomy using harmonic scalpel and electrocautery. Singap Med J.

[11] Khater A (2010). Harmonic scalpel as a single instrument in modified radical mastectomy. Is it more cost effective than electrocautery and ligature. Egypt J Surg.

[12] Sanguinetti A (2010). Ultrasound scissors versus electrocautery in axillary dissection: our experience. Il G Chir.

[13] Ribeiro GHFP (2013). Modified radical mastectomy: a pilot clinical trial comparing the use of conventional electric scalpel and harmonic scalpel. Int J Surg.

[14] Yilmaz KB (2011). Comparing scalpel, electrocautery and ultrasonic dissector effects: the impact on wound complications and pro-inflammatory cytokine levels in wound fluid from mastectomy patients. J Breast Cancer.

[15] Khan S, Chawla T, Murtaza G (2014). Harmonic scalpel versus electrocautery dissection in modified radical mastectomy: a randomized controlled trial. Ann Surg Oncol.

[16] Anlar B, Karaman N, Dogan L, Ozaslan C, Atalay C, Altinok M (2013). The effect of harmonic scalpel, electrocautery, and scalpel use on early wound complications after modified radical mastectomy. Eur Surg.

[17] Galatius H, Okholm M, Hoffmann J (2003). Mastectomy using ultrasonic dissection: effect on seroma formation. Breast Edinb Scotl.

[18] Iovino F, Auriemma PP, Ferraraccio F, Antoniol G, Barbarisi A (2012). Preventing seroma formation after axillary dissection for breast cancer: a randomized clinical trial. Am J Surg.

